# CircRNA PIP5K1A promotes the progression of glioma through upregulation of the TCF12/PI3K/AKT pathway by sponging miR-515-5p

**DOI:** 10.1186/s12935-020-01699-6

**Published:** 2021-01-07

**Authors:** Kebin Zheng, Haipeng Xie, Wensong Wu, Xichao Wen, Zhaomu Zeng, Yanfang Shi

**Affiliations:** Department of Neurosurgery, Affiliated Hospital of Hebei University, No.212Yuhua Road, Lianchi District, Baoding, 071000 Hebei China

**Keywords:** Glioma, Progression, CircPIP5K1A, miR-515-5p, TCF12, ceRNA

## Abstract

**Background:**

Increasing studies have revealed that circular RNAs (CircRNAs) make great contributions to regulating tumor progression. Therefore, we intended to explore the expression characteristics, function, and related mechanisms of a novel type of circRNA, PIP5K1A, in glioma.

**Methods:**

Firstly, reverse transcription-polymerase chain reaction (RT-PCR) was carried out to examine CircPIP5K1A expression in glioma tissues and adjacent normal tissues, and the correlation between CircPIP5K1A level and the clinical-pathological indicators of glioma was analyzed. Then, the CircPIP5K1A expression in various glioma cell lines was detected, and CircPIP5K1A overexpression and knockdown cell models were constructed. Subsequently, cell proliferation and viability were detected by the CCK8 method and BrdU staining. Cell apoptosis was detected by flow cytometry, and cell invasion was examined by Transwell assay. The expression of TCF12, PI3K/AKT pathway apoptotic related proteins (Caspase3, Bax, and Bcl2) and epithelial-mesenchymal transition (EMT) markers (E-cadherin, Vimentin, and N-cadherin) was determined by western blot or RT-PCR.

**Results:**

The results manifested that CircPIP5K1A was upregulated in glioma tissues (compared with that in normal adjacent tissues), and overexpressed CircPIP5K1A was related to glioma volume and histopathological grade. Functionally, overexpressing CircPIP5K1A notably elevated glioma cell proliferation, invasion, and EMT and inhibited apoptosis both in vivo and in vitro. Besides, CircPIP5K1A upregulated TCF12 and PI3K/AKT activation. Bioinformatics analysis testified that miR-515-5p was a common target of CircPIP5K1A and TCF12, while the dual-luciferase reporter assay and RNA immunoprecipitation (RIP) experiment further confirmed that CircPIP5K1A targeted miR-515-5p, which bound the 3′-untranslated region (UTR) of TCF12.

**Conclusions:**

Overall, the study illustrated that CircPIP5K1A is a potential prognostic marker in glioma and regulates glioma evolvement by modulating the miR-515-5p-mediated TCF12/PI3K/AKT axis.

## Background

Glioma is the most common tumor of the central nervous system with a high incidence and high malignancy [1]. Surgery, chemoradiotherapy, immunotherapy, and other comprehensive methods are currently used for its treatment clinically [2]. However, the overall therapeutic effects remain unsatisfactory due to the unclear pathogenesis and lack of specific treatment targets. In recent years, gene therapy for glioma has been widely studied, but the specific target and mechanism have not yet been well determined [3]. Hence, exploring the new molecular mechanism of glioma is of great value for its treatment and prognosis.

Circular RNAs (CircRNAs) are newly discovered circular noncoding RNAs involved in regulating gene expression at transcriptional and post-transcriptional levels [4]. Growing evidence shows that circRNAs play prominent roles in tumorigenesis and cancer development by regulating the downstream targeted miRNAs [5]. For example, circ-0006948 directly binds to miR-490-3p, which targets the 3′UTR of the oncogene high mobility group protein A2 (HMGA2) in esophageal squamous cell carcinoma (ESCC). Meanwhile, circ-0006948 enhances HMGA2 expression by sponging miR-490-3p, thus inducing EMT and aggravating ESCC [6]. In addition, circ-0037251 affects glioma cell proliferation and metastasis by regulating the miR-1229-3p/mTOR axis [7]. CircPIP5K1A is a newly discovered cancer-related circRNA, which is a powerful regulator in tumors. For instance, Zhang Qu et al. demonstrated that overexpressing circPIP5K1A enhances AP-1 expression and dampens the expression of IRF-4, CDX-2, and Zic-1 by downregulating miR-1273a, thus facilitating the migration and invasion of colon cancer [8]. Interestingly, the mechanism of action of circPIP5K1A in other diseases, including glioma, is rarely reported.

MicroRNAs (miRNAs) belong to the same class of noncoding RNAs as circRNAs, which are only 18 to 25 nucleotides in length. However, multiple miRNAs are found to regulate the pathological processes of many diseases through post-transcriptional gene silencing [9]. For instance, as a tumor suppressor, miR-187 attenuates cell growth and metastasis in glioma by dampening SMAD1 expression [10]. Besides, miR-214-3p abates EMT and migration of endometrial cancer (EC) cells by targeting TWIST1 [11]. As an essential member of the miRNAs, miR-515-5p is located at 19q13.42, and its pre-miRNA is 83 bp in length. In addition, it has been proved a key molecular in a variety of tumors. For example, miR-515-5p directly targets the 3′-UTR of TRIP13 and negatively regulates its expression, thereby acting as a tumor suppressor in prostate cancer [12]. Besides, miR-515-5p impedes tumor cells by targeting CXCL6 in non-small cell lung cancer (NSCLC) [13]. Nevertheless, the role of miR-515-5p in glioma needs further investigation.

Transcription factor 12 (TCF12) is a member of the helix-loop-helix protein family and serves either as an oncogene or tumor suppressor gene in multiple human cancers [14]. For example, TCF12 is a direct target of miR-26a, which has been shown to inhibit the growth of epithelial ovarian cancer (OC) and induce apoptosis by inhibiting the TCF12 expression [15]. In addition, Yang Jing et al. confirmed that TCF12 promotes the occurrence and development of hepatocellular carcinoma (HCC) by upregulating CXCR4 [16]. What’s more, PI3K is an intracellular phosphatidylinositol kinase, and AKT is a serine/threonine-specific protein. Existing studies have manifested that the PI3K/AKT signaling pathway is activated in most cancers, including glioma [17]. For example, matrine has anti-tumor effects in glioma by inducing apoptosis and autophagy and abating the PI3K/AKT and Wnt-β-catenin pathways [18]. Besides, some studies have found that overexpressing TCF12 upregulates p-AKT and p-PI3K and promotes gastric cancer (GC) development [19]. However, whether circPIP5K1A and TCF12 affect glioma by regulating the PI3K/AKT pathway remains elusive.

Here, we discovered that there is a targeted regulatory relationship between circPIP5K1A and miR-515-5p, miR-515-5p and TCF12 through bioinformatics. By detecting the expression of circPIP5K1A, miR-515-5p, TCF12 and PI3K/AKT in glioma tissues and cells and exploring the relationship among these molecules, we discovered that circPIP5K1A inhibits the miR-515-5p level and upregulates TCF12 by serving as a competitive endogenous RNA (ceRNA) of miR-515-5p, which in turn affects glioma cell proliferation and metastasis. In summary, this study aims to research the function of a novel circPIP5K1A/miR-515-5p/TCF12/PI3K/AKT axis in glioma, improve the study of its molecular mechanism, and provide referential molecular markers for clinical diagnosis and treatment for glioma.

## Methods

### Clinical specimen collection and processing

Forty-five cancerous and paired normal tissues of glioma patients who underwent resection in the Affiliated Hospital of Hebei University from March 2014 to March 2015 were selected. None of the patients received adjuvant treatment such as chemotherapy and radiotherapy before the surgery. The control specimens were obtained from the normal tissues of the same patient (at least 1 cm from the surgical margin), and no cancer cells were found through pathological examination. The glioma was diagnosed pathologically according to the World Health Organization (WHO) criteria. Immediately after removal, all specimens were stored in -196℃ liquid nitrogen until used for RNA extraction. The Ethics Committee of the Affiliated Hospital of Hebei University approved our study, and all of the involved patients signed informed consent.

### Cell culture

Human normal glial cell HEB and glioma cell lines (U87, TJ861, TJ905, U251, H4, and A172) were purchased from the American Type Culture Collection (ATCC, Rockville, MD, USA). The cells were cultured with RPMI1640 containing 10% fetal bovine serum (FBS) and 1% penicillin/streptomycin (Invitrogen, CA, USA)) at 37℃ and 5% CO_2_. RPMI1640 and FBS were provided by Thermo Fisher Scientific (MA, USA). During the logarithmic growth phase, cells were treated with 0.25% trypsin (Thermo Fisher, HyClone, Utah, USA) for trypsinization and passage. It was found that CircPIP5K1A was the lowest expressed in U87 cells and the highest expressed in A172. Therefore, U87 and A172 cells were chosen as the research object in subsequent studies.

### Cell transfection

CircPIP5K1A overexpression plasmids (pcDNA3.1-CircRNA PIP5K1A) and its small interfering RNA (Si-CircPIP5K1A), miR-515-5p mimics, TCF12 overexpression plasmids (pcDNA3.1-TCF12) and its small interfering RNA (Si-TCF12) and the corresponding negative controls were obtained from GenePharma (Shanghai, China). U87 and A172 cells were seeded in 24-well plates at 3 × 10^5^ cells/well and then incubated at 37℃ with 5% CO_2_ for 24 h before transfection using Lipofectamine® 3000 (Invitrogen; ThermoFisherScientific, Inc.). Reverse transcription-polymerase chain reaction (RT-PCR) was used to determine the transfection efficiency, and the cells were incubated at 37 °C with 5% CO_2_ for 24 h for further analysis.

### RT-PCR

Firstly, total RNA from tissues or cells was extracted using the TRIzol reagent (Invitrogen, Waltham, MA, USA). Then, Nanodrop-spectrophotometer was employed to measure RNA concentration and purity. Subsequently, we used a PrimeScript-RT Kit (Madison, WI, USA) to reverse-transcribe 1 µg of total RNA to synthesize its complementary DNA (cDNA), and then adopted SYBR® Premix-Ex-Taq™ (Takara, TX, USA) and ABI7300 system for RT-PCR according to the manufacturer's protocol. The total volume of the PCR system was 30 µL, and each sample contained 300 µg of cDNA. The amplification was initially performed at 95 °C for 10 min for 45 cycles. Namely, 95 °C for 10 s, 60 ℃ for 30 s, and 85 °C for 20 s. We converted all fluorescence data to relative quantification. β-actin was the internal reference of CircRNA PIP5K1A and TCF12, while U6 was that of miR-515-5p. RT-PCR was repeated three times. The primers were designed and synthesized by RiBo Biotechnology Co., Ltd (Guangzhou, China). CircPIP5K1A: forward primer 5′-AGATTCCCTAACCTCAACCAGA-3′, reverse primer 5′-CGAATGTTCTTGCCACCTGC-3′; TCF12: forward primer 5′-TCTGCCCCTAGATGAGACCT-3′, reverse primer 5′-GGCAATCATTCGGTCCTGTC-3′; miR-515-5p, forward primer: 5′-TTCTCCAAAAGAAAGCACTTTCTG-3, reverse primer 5′-CTCGCTTCGGCAGCACA-3′; GAPDH: forward primer 5′-TGATCTTCATGGTCGACGGT-3, reverse primer 5′-CCACGAGACCACCACCTACAACT-3′; U6, forward primer 5′-CTCGCTTCGGCAGCACA-3', reverse primer 5′-AACGCTTCACGAATTTGCGT-3'.

### RNase R assay

In RNase R assays, total cellular RNA (2.5 μg) was incubated with 10 U of RNase R (GeneSeed, Guangzhou, China) for 30 min at 37 °C, followed by the assessment of circPIP5K1A and GAPDH levels by RT-PCR.

### CCK 8 assay

CCK 8 assay was used to detect cell viability using a CCK8 kit (Beyotime, Shanghai, China). Firstly, U87 and A172 cell suspension (2 × 10^5^ cells/mL) were inoculated into 96-well plates with 100 μL/well. On the next day, the primary medium was removed, and the cell culture medium was supplemented. Subsequently, the medium was taken out after cultured in an incubator with 5% CO_2_ for 3, 6, 12, and 24 h, respectively. Finally, the CCK8 reagent was added at 10 μL/well and incubated for 2 h, and the OD value at the 450 nm wavelength was obtained by a microplate reader.

### BrdU assay

Firstly, cells at the logarithmic phase were supplemented with 10 μmol/L BrdU (Sigma, Shanghai, China). After DNA denaturation, the cells were incubated with the BrdU primary antibody (Abcam, ab6326,1:1000, CA, USA) at room temperature for 2 h. Then, fluorescent secondary antibodies were added and incubated for 2 h at room temperature. Later on, the nucleus was labeled by 10 μmol/L Hoechst33342. Finally, an inverted fluorescence microscope was used for imaging and statistical analysis.

### Transwell assay

Transwell chambers (Corting, NY, USA) were coated with 200 mg/mL matrigel (BD, SanJose, USA) and incubated overnight. Then, U87 and A172 cells (5 × 10^5^ mL) were suspended by the serum-free RPMI1640 medium, and 200 μL cell suspension was added into the upper chambers. Subsequently, RPMI1640 (500 μL) containing 10% FBS was placed in the lower chamber as a chemotactic agent. After incubation for 24 h, all uninvaded cells were removed. Then, matrigel membranes were fixed with paraformaldehyde and dyed with crystal violet buffer. At last, the number of invaded cells was counted by a phase-contrast microscope (Olympus, Tokyo, Japan). The experiment was repeated three times.

### RNA pull-down assay

The biotin-labeled circPIP5K1A probe was synthesized by BIOFAVOR Biotech (Wuhan, China). In brief, 2 × 10^7^ cells were harvested and lysed in 100 μL RIP lysis buffer on ice, then incubated with a high-affinity biotin-labeled probe for one hour at room temperature. Next, the suspension and streptavidin magnetic beads were mixed for one hour at room temperature. The beads were washed using RIP wash buffer, and the RNAs pulled down on the beads were extracted using TRIzol and analyzed by RT-PCR and gel electrophoresis.

### RNA immunoprecipitation (RIP) experiment

Magna RIP™ RNA-Binding Protein Immunoprecipitation Kit (Merck Millipore, USA) was used to conduct the RIP assay. 2 × 10^7^ U87 cells transfected with miR-505-5p or its negative control were collected and supplemented with 200 µL of RIP Lysis Buffer. Afterward, they were cleaved on ice for five min and centrifuged at 1500 rpm for 15 min to obtain the supernatant. Then, the extract was incubated with the anti-Ago2 or anti-IgG (Sigma) overnight. Subsequently, the supernatant was discarded after magnetic beads were washed with wash buffer five times, and then protease K lysate was added to the magnetic beads for lysis at 55 °C for 30 min. Finally, the supernatant was placed in a new centrifuge tube, and the total RNA was extracted by phenol–chloroform-isoamyl alcohol extraction and purified with isopropanol centrifugation. The levels of CircPIP5K1A and TCF12 were tested by RT-PCR.

### Dual-luciferase reporter assay

All luciferase reporter vectors (CircPIP5K1A-WT, CircPIP5K1A-MUT, TCF12-WT, and TCT12-MUT) were obtained from Promega (Madison, WI, USA). U87 cells (4.5 × 10^4^) were seeded in 48-well plates to 70% confluence. Then, U87 cells were co-transfected with miR-515-5p or its negative control with CircPIP5K1A-WT, CircPIP5K1A-MUT, TCF12-WT and TCF12-MUT using liposome 2000. Forty-eight hours after transfection, luciferase activity was determined following the manufacturer's guidelines. All experiments were made in triplicate.

### Western blot

The cells were collected and washed with cold PBS three times, and 100 ~ 200 μL RIPA lysate (Beyotime Biotechnology, Shanghai, China) was added to lyse the cells on ice. Then, the protein in the lysates was isolated through centrifugation, and the protein concentration was determined by the Bradford method. An equal amount of protein in each group was isolated on 10% SDS-PAGE, and then the proteins in the gel were transferred to PVDF membranes (Millipore, Bedford, MA, USA). Subsequently, the membranes were blocked at 4 °C for one hour by 5% BSA and incubated with the primary antibodies of anti-TCF12 antibody (ab70746, 1:1000, Abcam, MA, USA), anti-PI3K antibody (ab191606, 1:1000, Abcam, MA, USA), anti-PI3k (phosphoY607) antibody (ab182651, 1:1000, Abcam, MA, USA), anti-pan-AKT antibody (ab18785, 1:1000, Abcam, MA, USA), anti-AKT (phospho T308) antibody (ab38449, 1:1000, Abcam, MA, USA), anti-Bax antibody (ab32503, 1: 1000, Abcam,MA,USA), Anti-Bcl-2 antibody (ab59348, 1:1000, Abcam,MA,USA), Anti-Caspase 3 antibody (ab13847, 1:1000, Abcam, MA, USA), Anti-E-cadherin antibody (ab16505, 1:1000, Abcam, MA, USA), Anti-Vimentin-antibody (ab92547, 1:1000, Abcam, MA, USA) and Anti-N-cadherin antibody (ab18203,1:1000, Abcam, MA, USA) at 4 °C overnight. After being washed with TBST twice, the membranes were incubated with HRP-labeled Goat-anti-Rabbit secondary antibody (ab205718, 1:2500, Abcam) at room temperature for one hour. Finally, the membranes were washed three times, exposed with the ECL color reagent (Millipore, Bedford, MA, USA), and imaged with a scanner.

### Flow cytometry

After being treated with different factors, the cells were trypsinized and then collected through centrifugation (1500 rpm, 3 min). The harvested cells were treated with the cell apoptosis detection kit (Shanghai Aladdin Bio-Chem Technology Co., Ltd) as follows. First, the cells were washed with PBS twice. Then, 400 μL precooling PBS was added, and 10 μL of AnnexinV-FITC and 5 μL of PI were supplemented and incubated at 4 °C in the dark for 30 min. Immediately after that, flow cytometry was adopted to measure cell apoptosis, and the percentage of apoptotic cells was calculated after computer processing.

### Tumor formation assay in nude mice

Firstly, 4–6 week-old BALB/c-nu nude mice were selected to construct a tumor formation model. U87 and A172 cells in the logarithmic phase were chosen, and the cell concentration was adjusted to 2 × 10^8/^mL. Subsequently, 0.1 mL cell suspension was injected subcutaneously into the armpit of the left forelimb of each nude mouse. Each group had 10 mice. The survival rate and status of the mice were monitored, and the size and weight of the tumor in the newly dead mice were measured within 25 days after the injection.

### Statistical analysis

SPSS22.0 software (SPSS Inc., Chicago, IL, USA) was used for statistical analysis, and the results were presented as mean ± SD (*x* ± *s*). Overall survival and recurrence-free survival trends and curves were calculated by the Kaplan–Meier method, and differences were evaluated using the log-rank test. The measurement data between the two groups were compared by *t* test, and ANOVA test was used to compare the difference between multiple groups. *P* < 0.05 was considered statistically significant.

## Results

### CircPIP5K1A was highly expressed in glioma tissues and cells

**Fig. 1:**
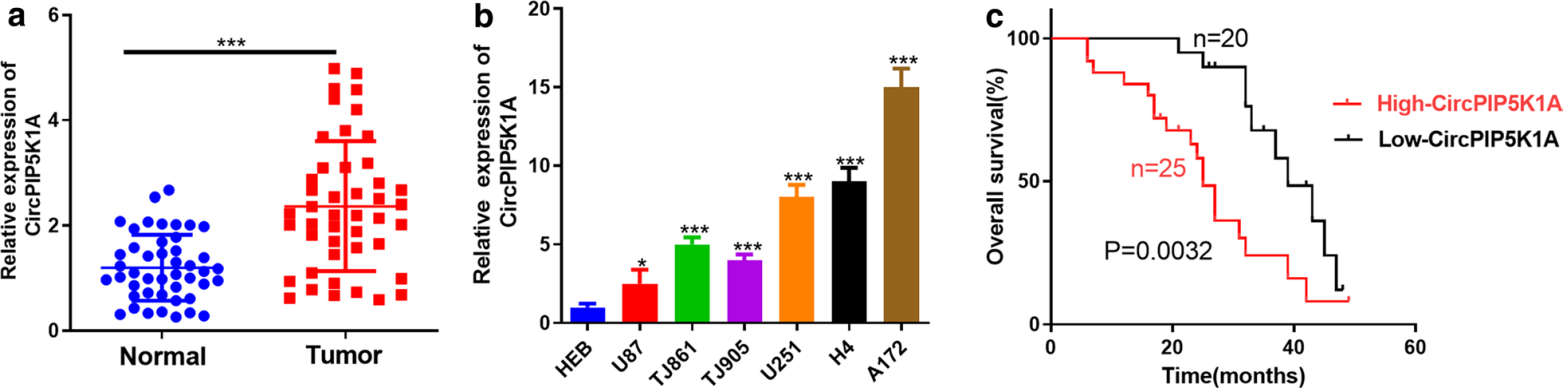
CircPIP5K1A expression in normal and cancerous tissues of glioma. **a** RT-PCR was used to verify the CircPIP5K1A expression in the tumorous and adjacent normal tissues. ***P < 0.0001. **b** RT-PCR was adopted to determine the circPIP5K1A expression in normal glioma cells HEB and tumor cell lines (U87, TJ861, TJ905, U251, H4, and A172), *P < 0.05, ***P < 0.001 vs. HEB group. **c** KM plotter was used for analyzing the relationship between circPIP5K1A expression with glioma prognosis. **d** CircPIP5K1A and GAPDH levels by RT-PCR in total cellular RNA incubated with RNase R

Firstly, we carried out RT-PCR to investigate the circPIP5K1A level in glioma tissues. It turned out that circPIP5K1A was upregulated compared with that in normal adjacent tissues (*P* < 0.05, Fig. 1a). In addition, the circPIP5K1A expression in different glioma cell lines was compared by RT-PCR, and the results manifested that it was obviously upregulated in glioma cell lines (U87, TJ861, TJ905, U251, H4, and A172) compared with that in normal human glial cells (HEB) (*P* < 0.05, Fig. 1b). Moreover, the survival time of glioma patients with high circPIP5K1A expression was shorter than that of with low CircPIP5K1A level, with larger tumor volume, higher tumor stage and Ki-67 rate (Fig. 1c and Table 1). To determine the stability of CircPIP5K1A, we then performed the RNase R assay. These data revealed that GAPDH transcript was decreased by RNase R digestion, and CircPIP5K1A was resistant to RNase R (*P* > 0.05, Additional file 1: Figure S1a, b). These results suggested that CircPIP5K1A is associated with the malignant phenotypes of glioma cells and is carcinogenic.

**Table 1:** Relationship between the CircPIP5K1A level and clinical characteristics in tissue samples from glioma patients

Characteristics	Patients	Expression of CircPIP5K1A		P-value
		Low-CircPIP5K1A	High-CircPIP5K1A	
Total	45	20	25	
Age (years)				
<45	20	8	12	0.592
≥45	25	12	13	
Gender				
Male	24	11	13	0.841
Female	21	9	12	
IDH1 mutation				
No mutation	24	10	14	0.8241
Mutation	19	9	11	
MGMT promoter methylation				
Unmethylation	26	13	12	0.5549
Methylation	23	10	13	
Tumor stage (WHO)				
I-II	27	11	16	0.014*
III-IV	18	4	14	
Ki-67 rate level				
Low	19	13	6	0.0057*
High	26	7	19	
Tumor volume				
<5 cm	28	15	11	0.036*
≥5 cm	17	5	14	

*P<0.05 was considered to be statically significant

### CircPIP5K1A affects proliferation, invasion, apoptosis and EMT of glioma cells

**Fig. 2:**
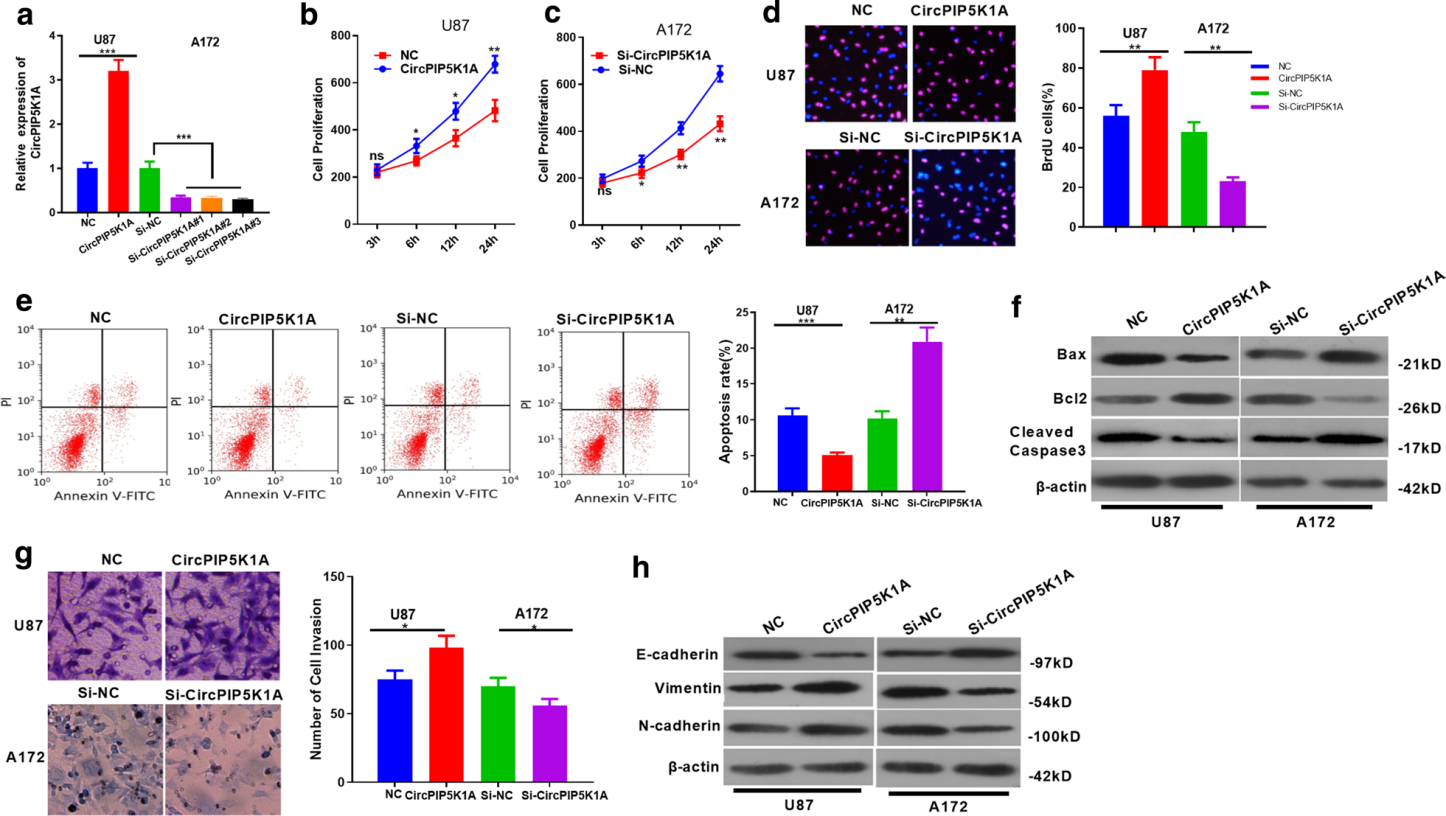
The influence of CircPIP5K1A on glioma cell proliferation, invasion, apoptosis and EMT. **a** CircPIP5K1A overexpression and knockdown models were constructed in glioma cell lines U87 and A172, respectively. B-D: CCK-8 method (**b**, **c**) and BrdU assay (**d**) were adopted to determine cell proliferation. **e**, **f** flow cytometry and western blot were conducted to detect the apoptosis of U87 and A172 cells. **g** Transwell assay was employed to determine the invasive ability of U87 and A172 cells. H: Western blot was employed to test EMT markers of E-cadherin, Vimentin and N-cadherin. *P < 0.05, **P < 0.01, ***P < 0.001

We constructed overexpression and knockdown models of circPIP5K1A in U87 and A172 to explore the effect of circPIP5K1A on glioma (*P* < 0.05, Fig. 2a). CCK8 and BrdU testified that cell proliferation and viability were significantly strengthened after circPIP5K1A overexpression, while the reverse effect was observed after circPIP5K1A knockdown (*P* < 0.05, Fig. 2b–d). Similarly, we employed flow cytometry and western blot to detect cell apoptosis. The results revealed that the apoptosis rate was dampened after CircPIP5K1A overexpression, but it was elevated after the circPIP5K1A knockdown (*P* < 0.05, Fig. 2e, f). Besides, the Transwell assay showed that overexpressing circPIP5K1A enhanced cell invasion, while knocking down circPIP5K1A weakened cell invasion (*P* < 0.05, Fig. 2g). Furthermore, we conducted western blot to detect the expression of EMT-related markers E-cadherin, Vimentin and N-cadherin. As shown in the figure, E-cadherin expression was notably attenuated after CircPIP5K1A overexpression, while Vimentin and N-cadherin expression was significantly elevated. In contrast, E-cadherin was upregulated, while Vimentin and N-cadherin were downregulated after circPIP5K1A knockdown (*P* < 0.05, Fig. 2h). These results demonstrated that circPIP5K1A is involved in glioma development by elevating tumor cell growth, invasion and EMT, and decreasing apoptosis.

### CircPIP5K1A promoted glioma growth and EMT in vivo

**Fig. 3:**
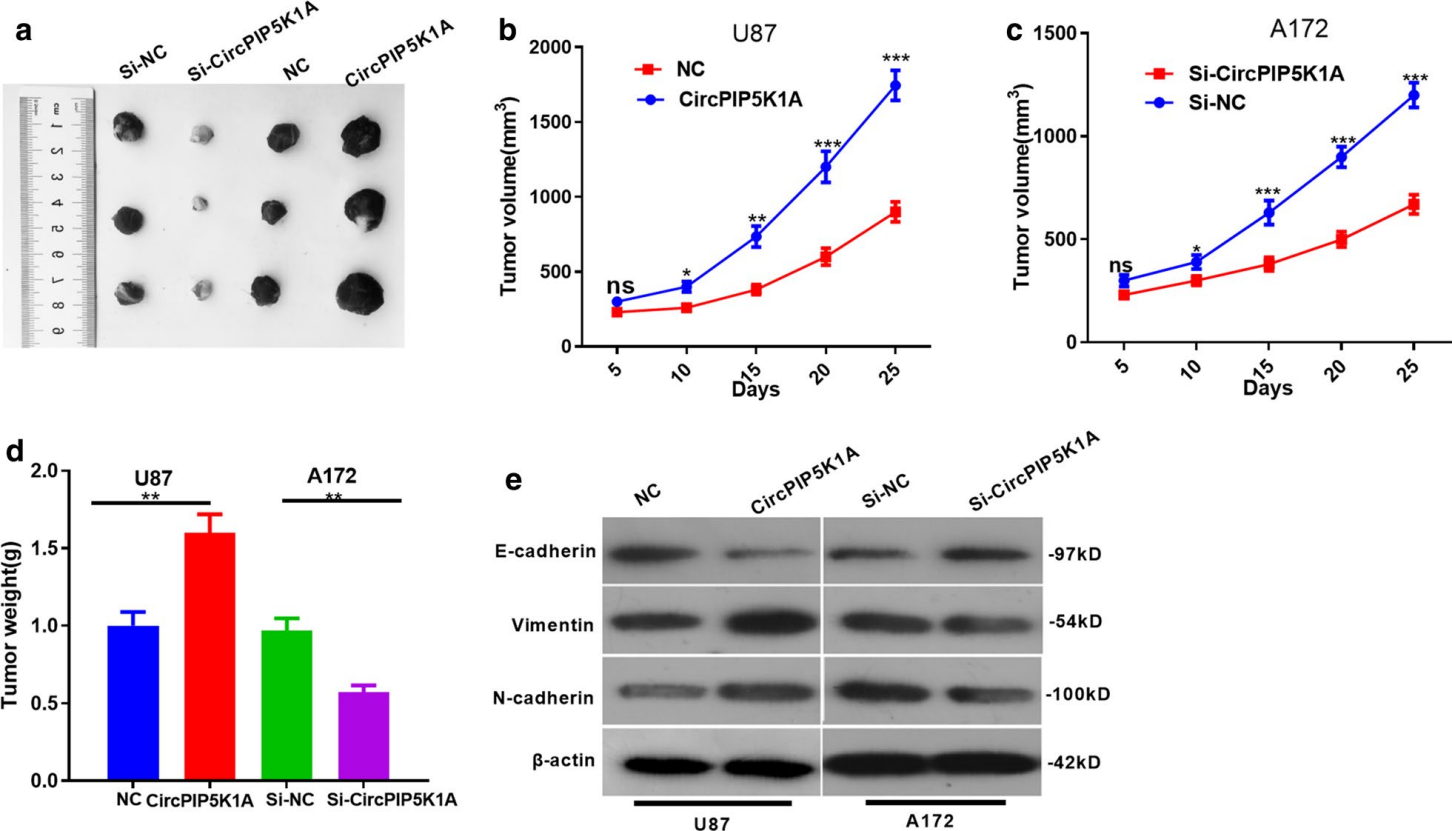
CircPIP5K1A promoted glioma growth and EMT transformation in vivo. **a** The nude mice were sacrificed 25 days later; the subcutaneous nodules were taken out and the image of tumors in each group was shown. **b**–**d** The volume and weight of the tumor nodules were calculated. **e** Western blot was employed to test the profiles of E-cadherin, Vimentin, and N-cadherin. NS *P* > 0.05, *P < 0.05, * *P < 0.01,***P < 0.001 vs.NC group or si-NC group

We constructed circPIP5K1A overexpression and knockdown cell lines in U87 and A172 respectively, and the tumor formation assay in nude mice was conducted to verify the effect of circPIP5K1A on glioma growth in vivo. We found that the tumor volume and weight were elevated by overexpressing circPIP5K1A, while they were dramatically reduced by knocking down circPIP5K1A (*P* < 0.05, Fig. 3a–d). In addition, western blot suggested that E-cadherin was downregulated, while Vimentin and N-cadherin were upregulated after circPIP5K1A overexpression. However, circPIP5K1A knockdown had the opposite effects (Fig. 3e). Hence, these results further confirmed that circPIP5K1A facilitates the growth and EMT of glioma cells.

### CircPIP5K1A promoted the expression of the TCF12 and PI3K/AKT signaling pathway activation

**Fig. 4:**
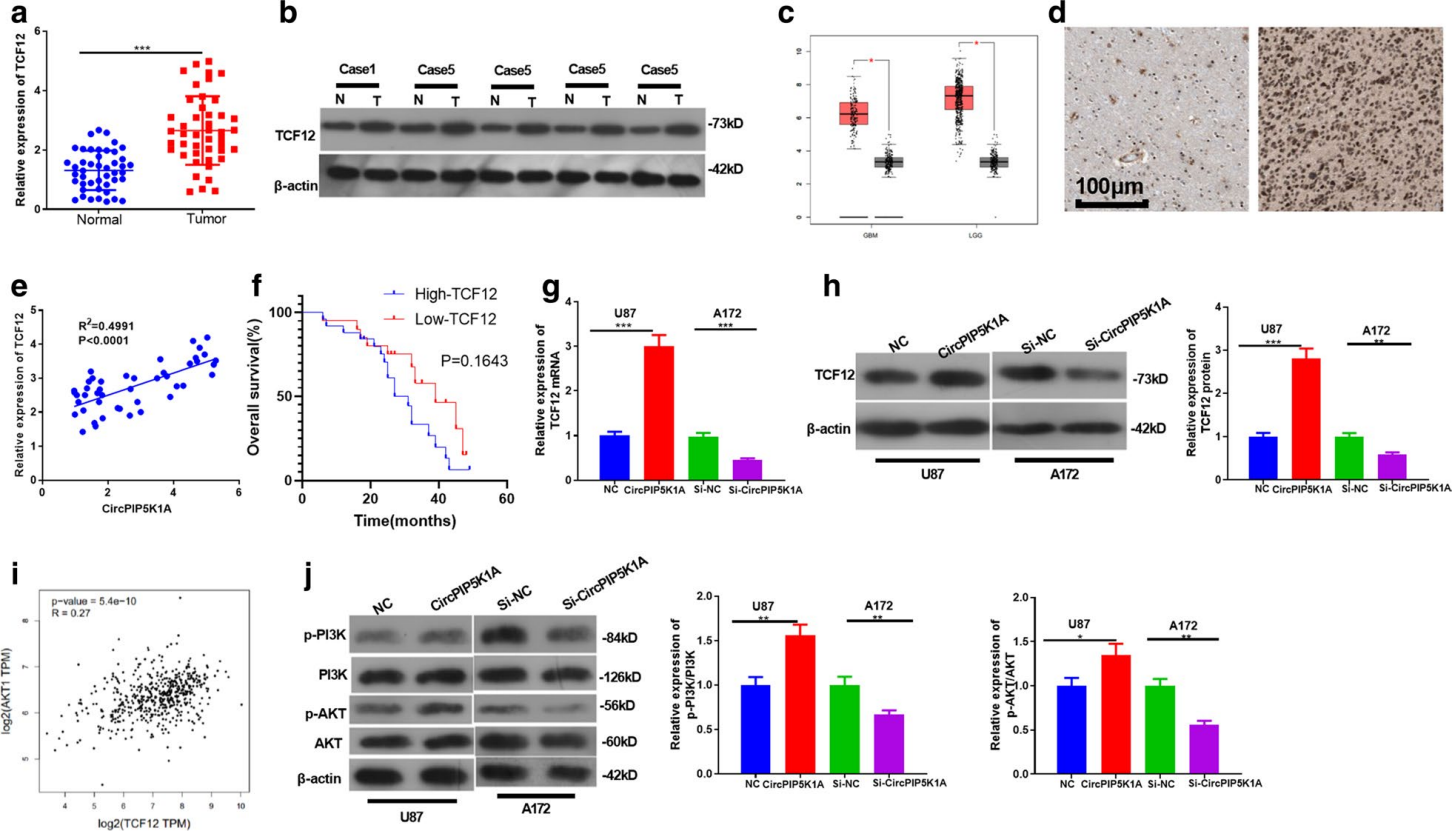
CircPIP5K1A promoted the expression of the TCF12 and PI3K/AKT signaling pathways. **a** TCF12 expression in glioma and adjacent normal tissues was detected by RT-PCR. **b** Protein expression of TCF12 in five cases of glioma and adjacent normal tissues was detected by western blot. **c** GEPIA (http://gepia.cancer-pku.cn/) was used for analyzing TCF12 expression in GBM and LGG. **d** TCF12 in glioma tissues and normal cerebral tissues was detected by IHC (data from The Human Protein Atlas (https://www.proteinatlas.org/). **e** Person correlation analysis detected the correlation between circPIP5K1A and TCF12 in the glioma tissues. **f**, **k**–**m** assay was used to analyze the relationship between TCF12 expression with glioma prognosis. **g**, **h** RT-PCR was performed to determine the expression of TCF12 mRNA (**g**) and protein (**h**). **i** GEPIA database showed a positive correlation between TCF12 and AKT1 in LGG. **j** Western blot was conducted to detect the PI3K/AKT activation. *P < 0.05, **P < 0.01, ***P < 0.001

We conducted western blot to explore the expression characteristics of TCF12 in tumors and found that TCF12 was upregulated in glioma tissues (compared with that in adjacent normal tissues (Fig. 4a, b). Besides, we discovered that TCF12 was upregulated both in GBM and LGG, as shown in GEPIA (http://gepia.cancer-pku.cn/) (Fig. 4c). On the other hand, TCF12 was overexpressed in glioma tissues, mainly distributed in the nucleus (Fig. 4d, data from The Human Protein Atlas (https://www.proteinatlas.org/)). Interestingly, there was a positive relationship between circPIP5K1A and TCF12 in glioma tissues (*R*^2^ = 0.499, *P* < 0.0001, Fig. 4e). We then analyzed the relationship between TCF12 expression with glioma prognosis. It was found that glioma patients with higher level of TCCF12 had poorer survival than those with lower level of TCF12 (*P* > 0.05, Fig. 4f). In the cell model, upregulating circPIP5K1A increased TCF12 expression (Fig. 4g, h). Moreover, we found that TCF12 was positively related to AKT1 in LGG through GEPIA database analysis (http://gepia.cancer-pku.cn/) (Fig. 4i). Furthermore, western blot was employed to detect the effect of CircPIP5K1A on the PI3K/AKT signaling pathway. The results testified that overexpressing CircPIP5K1A elevated the expression of p-PI3K and p-AKT, while knocking down CircPIP5K1A resulted in the opposite effect (*P* < 0.05, Fig. 4j). These results indicated that TCF12 and PI3K/AKT were positively regulated by circPIP5K1A.

### Overexpressing TCF12 aggravated the malignant phenotypes of glioma cells

**Fig. 5:**
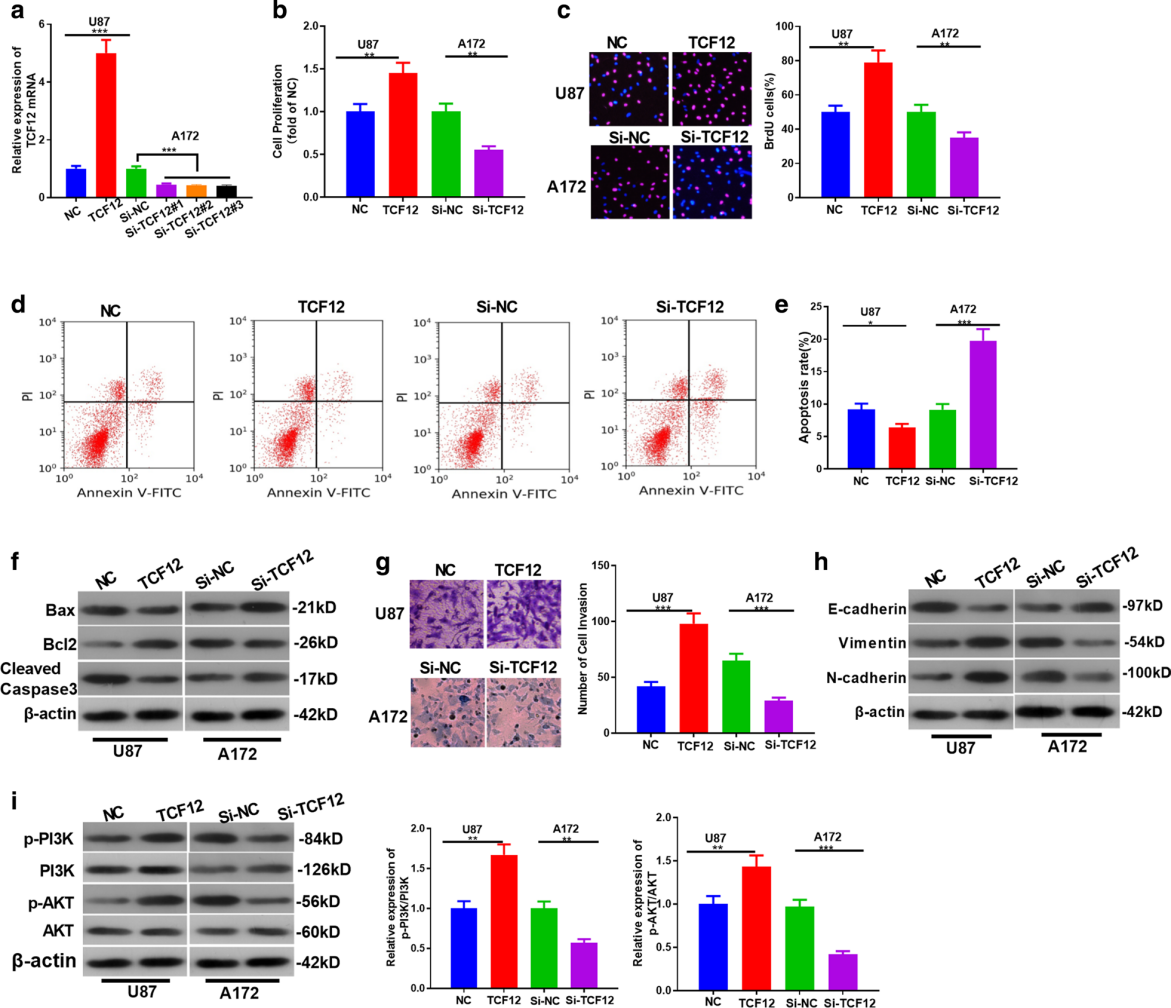
Overexpressing TCF12 promoted the malignant phenotypes of glioma cells. **a** Overexpression and knockdown models of TCF12 were constructed in U87 and A172, respectively, and the TCF12 expression was detected by western blot. B-C: CCK8 (**b**) and BrdU assay (**c**) were employed to detect cell proliferation. **e**–**g** Flow cytometry and western blot were used to verify the effect of TCF12 regulation on apoptosis. **g** Transwell assay was conducted to examine the effect of TCF12 regulation on cell invasion. **h** Western blot was carried out to measure the effect of TCF12 regulation on EMT. **i** The effect of TCF12 on PI3K/AKT was determined by western blot. *P < 0.05, **P < 0.01, ***P < 0.001

We conducted a gain- and loss-of functions assay of TCF12 in U87 and A172 respectively to verify the influence of TCF12 on glioma (*P* < 0.05, Fig. 5a). In addition, CCK8 and BrdU were implemented to detect cell growth and viability. It was confirmed that overexpressing TCF12 enhanced cell proliferation and viability, while knocking down TCF12 led to the opposite effects (*P* < 0.05, Fig. 5b–d). Besides, flow cytometry and western blot certified that the apoptosis rate was notably dampened after TCF12 overexpression, while it was dramatically increased after TCF12 knockdown compared with that of the NC group (*P* < 0.05, Fig. 5e, f). Further, a Transwell assay was carried out to examine the effect of TCF12 regulation on cell invasion. As shown in the figure, the cell invasion was significantly enhanced after TCF12 overexpression, while it was obviously inhibited after TCF12 knockdown (*P* < 0.05, Fig. 5g). Furthermore, the Transwell assay was employed to investigate the TCF12 regulation on E-cadherin, Vimentin and N-cadherin in glioma cells. The results revealed that TCF12 overexpression repressed E-cadherin expression but upregulated Vimentin and N-cadherin. Conversely, knocking down TCF12 increased E-cadherin level but inhibited the expression of Vimentin and N-cadherin (Fig. 5h). Similarly, western blot was conducted to detect the activation of the PI3K/AKT signaling pathway. The results manifested that overexpressing TCF12 promoted the expression of p-PI3K and p-AKT compared with that of the NC group, while knocking down TCF12 showed the opposite result (*P* < 0.05, Fig. 5i). The above results indicated that overexpressing TCF12 elevated the proliferation, invasion and EMT and weakened apoptosis of glioma cells, and enhanced the PI3K/AKT expression.

### MiR-515-5p shared the target with CircPIP5K1A and TCF12

**Fig. 6:**
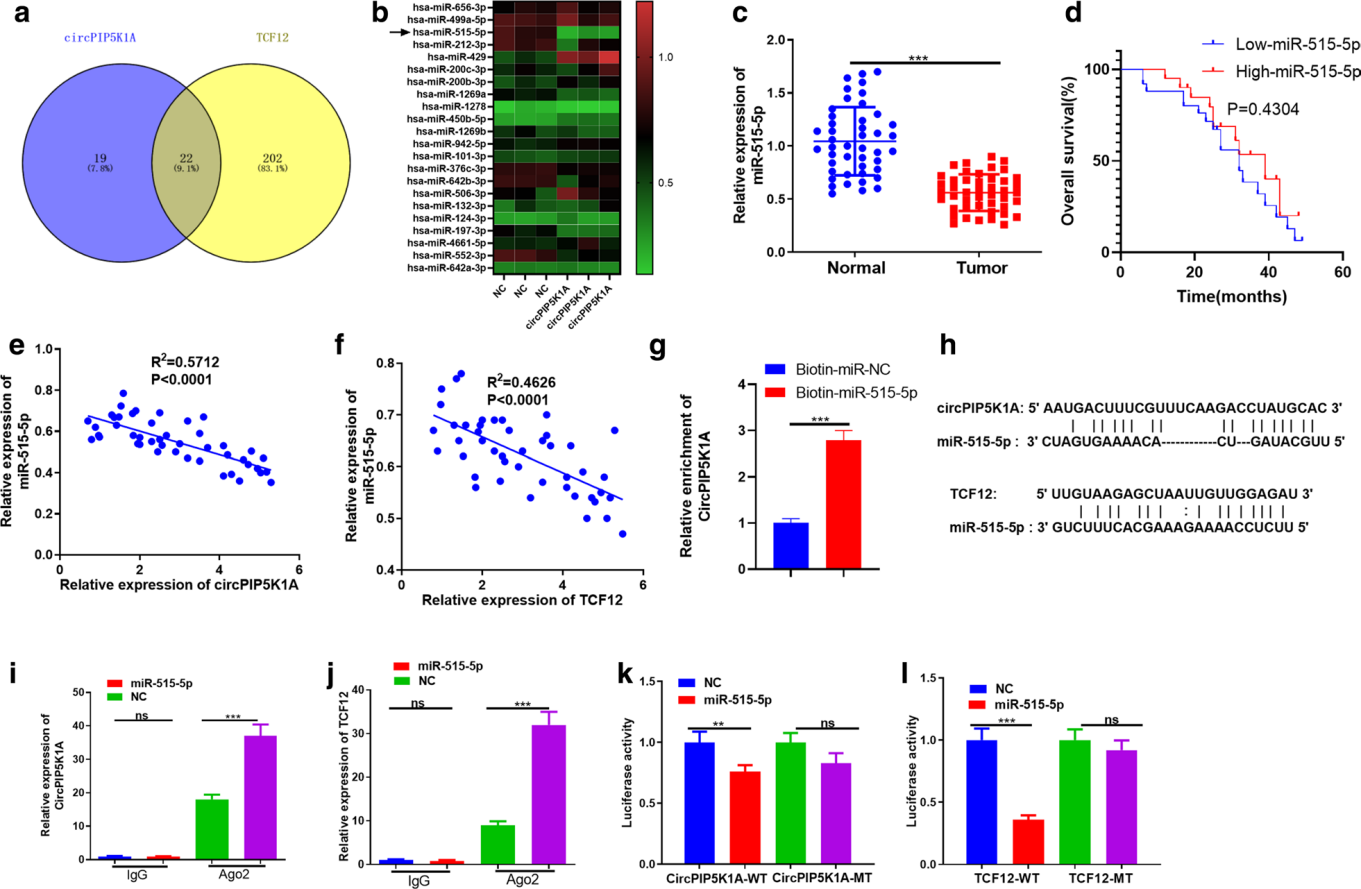
The targeted relationships among circPIP5K1A, TCF12 and miR-515-5p. **a** The miRNA target of circPIP5K1A and TCF12 was analyzed through Starbase, and a total of 22 miRNAs were common targets of CircPIP5K1A and TCF12. **b** RT-PCR was used to determine the levels of the 22 miRNAs in circPIP5K1A overexpressed cell. **c** The miR-515-5p expression in glioma tissues and adjacent normal tissues was detected by RT-PCR. **d** K-M assay was used to analyze the relationship between miR-515-5p expression with glioma prognosis. **e**, **f** Person correlation analysis was adopted to detect the correlation between miR-515-5p and circPIP5K1A, miR-515-5p and TCF12 in the tissues of glioma patients. **g** Relative circPIP5K1A level in U87 cells lysates captured by biotin-labeled miR-515-5p or NC was detected by RNA pull-down assay. H: The binding relationships of miR-515-5p with both circPIP5K1A and TCF12 was shown. **i**, **j** RIP assay was employed to examine the bindings of CircPIP5K1A and TCF12 with ant-Ago2 antibody in U87 cells. **k**, **l** Dual-luciferase reporter assay was analyzed to clarify the targeting binding relationships between miR-515-5p and circPIP5K1A, miR-515-5p and TCF12 in U87 cells. Ns *P* > 0.05, **P < 0.01, ***P < 0.001

Inspired by the circRNA-miRNA-mRNA regulatory network, we explored the miRNA target of circPIP5K1A and TCF12 through Starbase (http://starbase.sysu.edu.cn/). The results showed that 22 miRNAs were common targets of CircPIP5K1A and TCF12 (Fig. 6a). Next, we used RT-PCR to determine the levels of these 22 miRNAs in circPIP5K1A overexpressed cells. It was found that miR-515-5p was most significantly downregulated (Fig. 6b). Next, the miR-515-5p level in glioma tissues was detected by RT-PCR, and it was found to be obviously reduced in glioma tissues compared with that in the adjacent normal tissues (*P* < 0.05, Fig. 6c). When analyzing the prognostic value of miR-515-5p in glioma, we found there was no significant difference between glioma patients’ overall survival and miR-515-5p level (*P* > 0.05, Fig. 6d). In addition, Person correlation analysis revealed that circPIP5K1A and miR-515-5p was negatively correlated in glioma cells (*R*^2^ = 0.571, *P* < 0.001, Fig. 6e), which was the same of TCF12 and miR-515-5p (*R*^2^ = 0.463, *P* < 0.001, Fig. 6f). The binding relationship of miR-515-5p with both circPIP5K1A and TCF12 are shown in Fig. 6f. We conducted the following experiments to clarify the targeting relationship among the three molecules. First, we used biotin-labeled miR-515-5p mimics and NC to capture circPIP5K1A and found that more circPIP5K1A was captured by miR-515-5p mimic (*P* < 0.05, Fig. 6g). The binding sites between circPIP5K1A and miR-515-5p, miR-515-5p and TCF12 were shown in Fig. 6h. Next, we performed the RIP assay and dual-luciferase reporter gene assay. The results revealed that the amount of precipitated circPIP5K1A and TCF12 in the Ago2 antibody group was significantly higher than that in the IgG group after miR-515-5p transfection, suggesting that circPIP5K1A and TCF12 bound to Ago2 through miR-515-5p (*P* < 0.05, Fig. 6i, j). Furthermore, miR-515-5p markedly inhibited the luciferase activity of circPIP5K1A-WT and TCF12-WT while had no effect on circPIP5K1A-MUT and TCF12-MUT (*P* < 0.05, Fig. 6k, l). These results illustrated that miR-515-5p was a downstream target of circPIP5K1A and an upstream target of TCF12.

### CircPIP5K1A regulated the TCF12/PI3K/Akt expression by sponging miR-515-5p

**Fig. 7:**
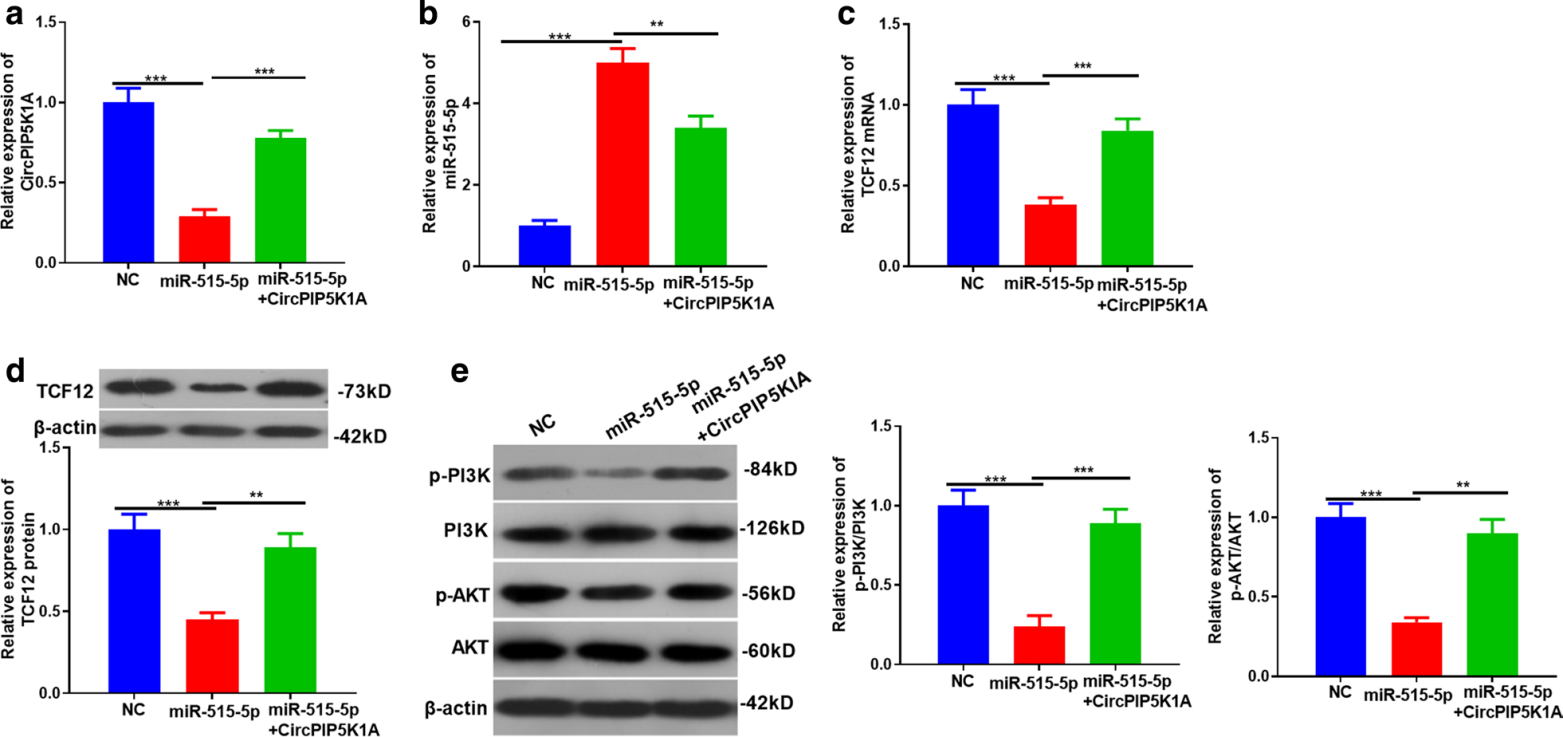
CircPIP5K1A regulated TCF12/PI3K/AKT expression by sponging miR-515-5p. **a**–**c** RT-PCR was carried out to detect the expression of CircPIP5K1A, miR-515-5p and TCF12 mRNA in U87 cells after transfecting circPIP5K1A overexpression plasmids or miR-515-5p mimics. **d**, **e** Western blot was constructed to monitor the expression of TCF12 and PI3K/AKT. **P < 0.01, ***P < 0.001

The rescue experiment was conducted to verify whether there was a regulatory axis of circPIP5K1A/miR-515-5p/TCF12/PI3K/Akt in glioma. It turned out that circPIP5K1A was downregulated, while miR-515-5p was upregulated in the miR-515-5p mimic group. On the other hand, circPIP5K1A was upregulated, while miR-515-5p was downregulated after CircPIP5K1A overexpression compared with the miR-515-5p group (*P* < 0.05, Fig. 7a, b). Next, we conducted RT-PCR and western blot to monitor changes in the TCF12 level. The results manifested that TCF12 mRNA and protein were downregulated by miR-515-5p overexpression, while supplementing circPIP5K1A enhanced the TCF level (*P* < 0.05, Fig. 7c, d). Further, we used western blot to test the activation of the PI3K/AKT signaling pathway. The results illustrated that upregulating miR-515-5p inhibited the levels of p-PI3K and p-AKT compared with NC, while supplementing circPIP5K1A in the miR-515-5p group increased p-PI3K and p-AKT expression (*P* < 0.05, Fig. 7e). The above results suggested that circPIP5K1A regulated TCF12 by sponging miR-515-5p, thereby activating the PI3K/AKT pathway.

## Discussion

Here, we explored a novel circPIP5K1A in the development of glioma. Our data suggested that circPIP5K1A is upregulated in glioma and predicts worse survival of glioma patients. Further, circPIP5K1A targeted miR-515-5p, thus upregulating the TCF12-PI3K/AKT axis.

CircRNAs are involved in modulating the occurrence and development of multiple tumors, including glioma [20]. For example, circSMO742 promotes glioma by sponging miR-338-3p to regulate SMO expression [21]. In addition, Ding Chenyu et al. showed that circNFIX abates glioma cell proliferation and metastasis by upregulating miR-378e and inhibiting RPN2 expression [22]. Meanwhile, accumulating evidence has confirmed that circPIP5K1A is a powerful regulator in different types of cancers. For instance, circPIP5K1A is upregulated in ovarian cancer and inhibits the migration, proliferation, and invasion of ovarian cancer cells [23]. Additionally, it has been demonstrated that circPIP5K1A promotes NSCLC proliferation and metastasis by upregulating HIF-1α [24]. In view of the above studies, we speculated that circPIP5K1A also plays a vital regulatory role in glioma. Interestingly, we found that circPIP5K1A is overexpressed in glioma tissues and cell lines, and our in vivo and in vitro experiments confirm that circPIP5K1A promotes the proliferation, invasion and EMT while attenuated the apoptosis of glioma cells, suggesting that circPIP5K1A functions as a prognostic factor and a therapy target in glioma.

Previously, abundant studies have reported that miR-515-5p is a powerful tumor suppressor. For example, miR-515-5p, negatively regulated by LINC00673, is downregulated in breast cancer, and it exerts an anti-tumor effect by downregulating the MAPK4/Hippo signaling pathway [25]. Similarly, miR-515-5p dampens the proliferation and metastasis of prostate cancer by targeting TRIP13 [12]. The results of this study also revealed that miR-515-5p was downregulated in glioma tissues and cell lines and negatively correlated with circPIP5K1A expression, which was consistent with the above reports on the anti-tumor effect of miR-515-5p.

Growing studies have found that circRNAs act as the ceRNAs of miRNA. For example, circZNF609 sponges miR-134-5p to promote BTG-2 expression as a ceRNA, thus weakening proliferation and migration of glioma cells [26]. In addition, overexpressing circPCMTD1 downregulates miR-224-5p and upregulates mTOR, thus aggravating glioma [27]. Here, we found a binding site between circPIP5K1A and miR-515-5p through StarBase. Combined with the above studies, we hypothesized that miR-515-5p might act as a downstream molecule of circPIP5K1A in glioma. Next, we verified the targeting relationship between the two by the RIP and dual-luciferase reporter gene assay. Furthermore, overexpressing circPIP5K1A dampened the inhibitory effects of miR-515-5p. Therefore, circPIP5K1A exerted its biofunctions by sponging miR-515-5p.

TCF12 is reported to be involved in modulating cell growth and differentiation and is carcinogenic in multiple malignant tumors [16]. For example, downregulation of TCF12 dampens the growth, migration and invasion of ovarian cancer and promotes apoptosis [28]. In addition, Shu Longwen et al. showed that overexpressing TCF12 attenuates the inhibitory effect of miR-204 on cervical cancer cell metastasis [29]. Surprisingly, TCF12 was confirmed to activate the PI3K/AKT signaling pathway to affect tumor progression. For example, TCF12 expedites hepatocellular carcinoma (HCC) by upregulating CXCR4 and its ligand CXCL12 and activating the MAPK/ERK and PI3K/AKT pathways [30]. Besides, Wang Xuekui et al. found that TCF12 accelerates gastric cancer development by targeting miR-183 and activating PI3K/AKT [19]. Here, we have confirmed through in vitro experiments that overexpressing TCF12 promotes cell proliferation, invasion and EMT, weakens apoptosis, and activates the PI3K/AKT signaling pathway in glioma. Through the bioinformatics database, the targeted binding relationship between miR-515-5p and TCF12 was analyzed, which prompted us to further explore whether circPIP5K1A plays a carcinogenic role by indirectly upregulating TCF12 through miR-515-5p. Moreover, miR-515-5p negatively regulated TCF12 expression. Meanwhile, circPIP5K1A positively modulated TCF12 and the PI3K/AKT pathway, which was consistent with Song H et al.'s report in 2020 that circPIP5KIA activated the PI3K/AKT pathway in gastric cancer [31]. These results suggested that circPIP5K1A plays a biological role by modulating TCF12-PI3K/AKT, which is consistent with our previous hypothesis that circPIP5K1A promotes glioma progression through the miR-515-5p-TCF12-PI3K/AKT axis.

Overall, circPIP5K1A facilitates glioma cell proliferation, metastasis and EMT, and inhibits apoptosis by targeting the miR-515-5p-TCF12-PI3K/AKT axis. The result provides a better understanding of gene-targeted therapy and the prognosis of glioma. However, the number of experimental cases in this study may be insufficient. It is necessary to clarify the expression characteristics of genes in more clinical samples or further study the effects of genes in clinical trials. Moreover, in vivo experiments were needed to further investigate the circPIP5K1A-miR-515-5p-TCF12-PI3K/AKT axis in glioma progresson.

## Supplementary Information

**Additional file 1: Figure S1.** The purification of circPIP5K1A. The RNase R digestion experiment was used to purify circPIP5K1A in U87 and A172 cells.

## Data Availability

The data sets used and analyzed during the current study are available from the corresponding author on reasonable request.
