# Supplementary figures and images for: CircRNA PIP5K1A promotes the progression of glioma through upregulation of the TCF12/PI3K/AKT pathway by sponging miR-515-5p
Source: Cancer Cell Int. 2021 Jan 7;21:27. doi: 10.1186/s12935-020-01699-6 (PMC7789671; doi:10.1186/s12935-020-01699-6)

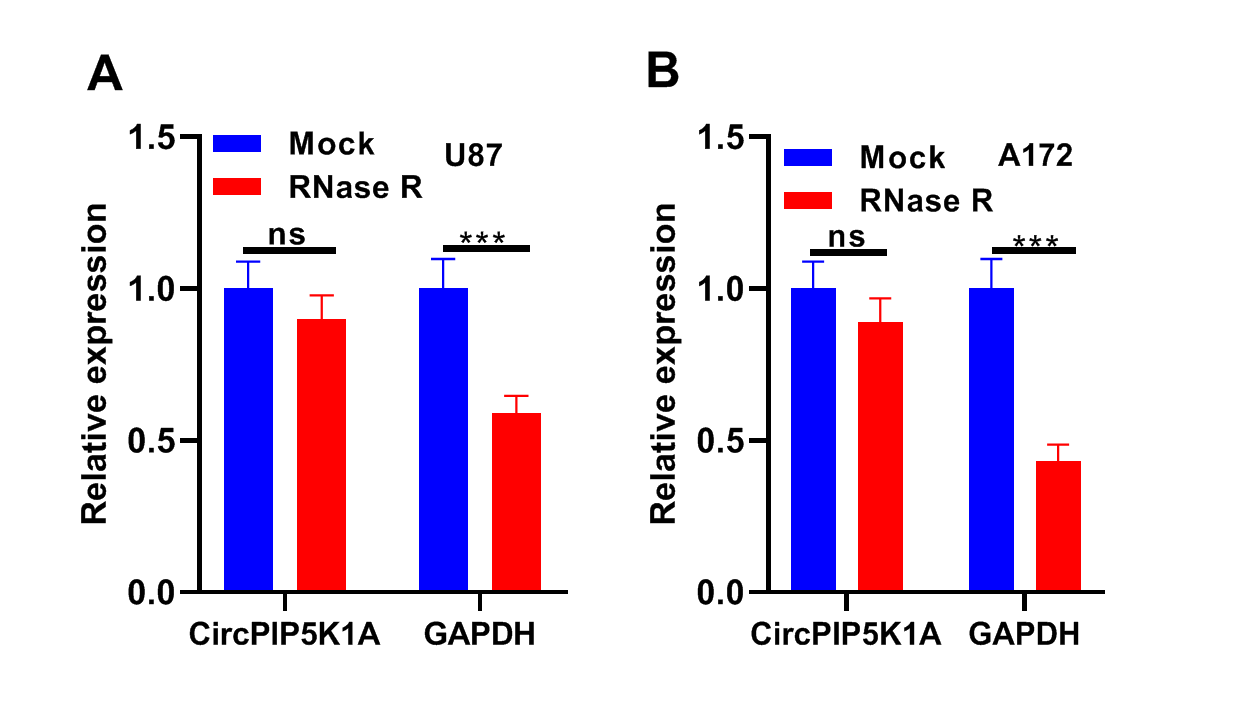

Supplement: Supplementary file 1 — Additional file 1: Figure S1. The purification of circPIP5K1A. The RNase R digestion experiment was used to purify circPIP5K1A in U87 and A172 cells. [file 12935_2020_1699_MOESM1_ESM.tif]
